# Intestinal Lymphatic Endothelial Cells Produce R-Spondin3

**DOI:** 10.1038/s41598-018-29100-7

**Published:** 2018-07-16

**Authors:** Reiki Ogasawara, Daigo Hashimoto, Shunsuke Kimura, Eiko Hayase, Takahide Ara, Shuichiro Takahashi, Hiroyuki Ohigashi, Kosuke Yoshioka, Takahiro Tateno, Emi Yokoyama, Ko Ebata, Takeshi Kondo, Junichi Sugita, Masahiro Onozawa, Toshihiko Iwanaga, Takanori Teshima

**Affiliations:** 1 Department of Hematology, Hokkaido University Faculty of Medicine and Graduate School of Medicine, Sapporo, Japan; 2 Laboratory of Histology and Cytology, Department of Anatomy, Hokkaido University Graduate School of Medicine, Sapporo, Japan

## Abstract

The R-Spondin (R-Spo) family regulates WNT signaling and stimulates the proliferation and differentiation of intestinal stem cells (ISCs). R-Spo plays a critical role in maintaining intestinal homeostasis, but endogenous producers of R-Spo in the intestine remain to be investigated. We found that R-Spo3 was the major R-Spo family member produced in the intestine and it was predominantly produced by CD45^−^CD90^+^CD31^+^ lymphatic endothelial cells (LECs) in the lamina propria of the intestinal mucosa. Transcriptome analysis demonstrated that LECs highly expressed R-Spo receptor, *Lgr5*, suggesting an autocrine stimulatory loop in LECs. LECs were significantly reduced in number, and their R-Spo3 production was impaired in intestinal graft-versus-host disease (GVHD) after allogeneic hematopoietic stem cell transplantation. The impaired production of R-Spo3 in the intestine may be a novel mechanism of delayed tissue repair and defective mucosal defense in intestinal GVHD. We demonstrate a novel role of intestinal LECs in producing R-Spondin3 to maintain intestinal homeostasis.

## Introduction

The intestinal mucosal epithelium is composed of a single layer of intestinal epithelial cells, including absorptive enterocytes, Paneth cells, goblet cells, enteroendocrine cells, and Tuft cells. All intestinal epithelial cells originate from leucine-rich repeat-containing G-protein-coupled receptor 5 (Lgr5) expressing intestinal stem cells (ISCs)^1,2^. Enterocytes have a rapid turnover time of 3–5 days; loss of ISCs in inflammatory diseases results in disrupts of barrier function of the intestinal mucosa and allows systemic translocation of intraluminal pathogens, exacerbating the underlying diseases^3–5^.

The R-Spondin (R-Spo) family is the ligand of Lgr5 on ISCs and potentiates WNT/β-catenin signaling by inhibiting the turnover of WNT receptors. The R-Spo protein family in mammals consists of four members with a similar structure: R-Spo1, R-Spo2, R-Spo3, and R-Spo4^6,7^. We and others have shown that administration of recombinant human R-Spo1 promotes Lgr5^+^ ISCs to self-renew and differentiate towards enterocytes, Paneth cells, and goblet cells^4,8,9^. R-Spos are also indispensable for *in vitro* culture of intestinal organoids^1^. It has been shown that R-Spo3 and, to a lesser extent, R-Spo2 play a critical role in tissue regeneration after radiation-induced mucosal injury in the gut using neutralizing antibodies against each member of the R-Spo family^10^. However, it remains to be elucidated which cells in the small intestine produce R-Spos. We investigated the producers of R-Spos in the murine small intestine and found that R-Spo3 was produced by CD45^−^CD31^+^CD90^+^ lymphatic endothelial cells (LECs) in the lamina propria. Interestingly, the numbers of LECs and R-Spo3 production were reduced in mouse graft-versus-host-disease (GVHD) after allogeneic hematopoietic stem cell transplantation (SCT).

## Results

### R-Spo3 is the major R-Spo protein produced in the intestine

**Figure 1:**
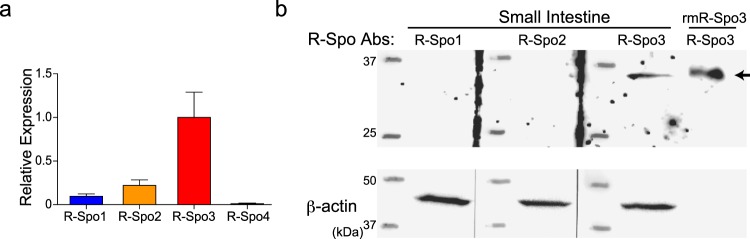
R-Spo3 is the major R-Spo molecule produced in the intestine. (**a**) mRNA was extracted from the small intestine of naïve B6D2F1 mice and subjected to quantitative PCR analysis of R-Spo family genes. Relative expressions of *R-Spo 1-4* normalized to *18S rRNA* from one of two independent experiments with similar results is shown as the means ± SE of n-fold difference relative to the expression of R-Spo3 (n = 6/group). (**b**) R-Spo1, R-Spo2, and R-Spo3 proteins as well as β-actin as a loading control in the lysates of small intestines of naïve B6D2F1 mice were detected by western blotting. Cropped images for R-Spo bands (top), and β-actin bands taken with shorter exposure time than that for R-Spondins (bottom) are shown. Images of full length gel with ranged exposure times are shown in Supplementary Figure 1. Recombinant mouse R-Spo3 (rm-R-Spo3) was applied on the far-right lane as a control (arrow).

First, we investigated which members of the R-Spo family were endogenously produced in the intestine. mRNA was extracted from the small intestine of naïve B6D2F1 mice and subjected to quantitative PCR analysis of R-Spo family genes. We found that *R-Spo3* and, to a lesser extent, *R-Spo1* and *R-Spo2* were expressed in the small intestine, while *R-Spo4* was not detected (Fig. 1a). To confirm the production of R-Spos at the protein level, lysates of the small intestine of naïve B6D2F1 mice were subjected to western blotting. Significant expression of R-Spo3, but not R-Spo1 or R-Spo2, was detected in the lysates, indicating that R-Spo3 is the major R-Spo protein produced in the small intestine (Fig. 1b, Supplementary Figure 1).

### CD45^−^CD90^+^CD31^+^ lymphatic endothelial cells in the intestine produce R-Spo3

**Figure 2:**
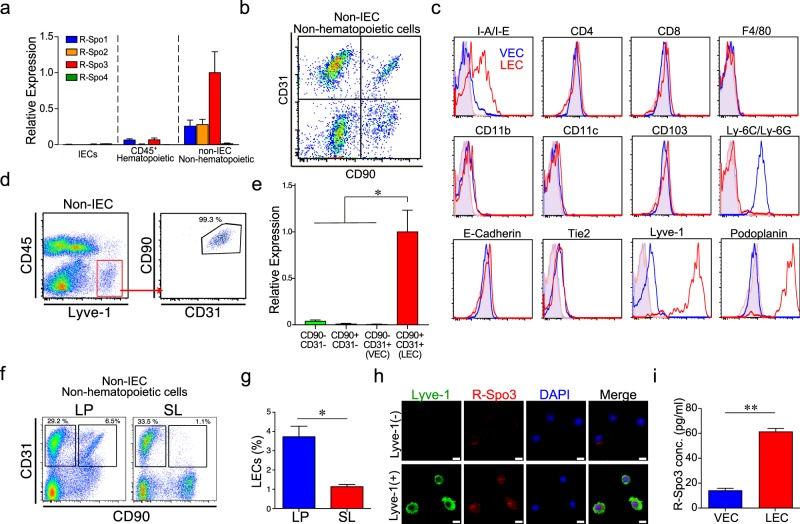
CD45^−^CD90^+^CD31^+^ LECs in the small intestine produce R-Spo3. (**a**) After IECs were harvested from the small intestine of naïve B6D2F1 mice by incubating with EDTA, CD45^+^ hematopoietic cells and CD45^−^ non-IEC non-hematopoietic cells were purified. The relative expressions of *R-Spo* genes normalized to that of *18S rRNA* in each population was measured by quantitative PCR. Data from two independent experiments with similar results were combined and shown as the means ± SE of n-fold difference relative to the expression of R-Spo3 of non-IEC non-hematopoietic cells (n = 12/group). (**b**) Representative FACS plot of CD31 and CD90 expression on CD45^−^ cells from the small intestine. (**c**) Flow cytometric analysis of surface markers on CD45^−^CD31^+^CD90^−^ VECs (blue histograms) and CD45^−^CD31^+^CD90^+^ LECs (red histograms) from the small intestines. Shaded histograms represent unstained controls. (**d**) Expression of CD31 and CD90 was evaluated on gated CD45^−^Lyve-1^+^ cells in the small intestine. (**e**) Expression levels of R-Spo3 in each population shown in (**b**). Data from one of two independent experiments with similar results are shown as the means ± SE (n = 4/group). (**f**,**g**) Lamina propria (LP) and the serosal layer (SL) of the small intestine were mechanically separated. Representative dot plots (**f**) and percentage (**g**) of LECs among CD45^−^ non-IEC non-hematopoietic cells are shown. Data from two independent experiments with similar results were combined and shown as the means ± SE in (**f**) (n = 6/group). (**h**) LECs were sorted from the small intestines. Immunofluorescent staining of Lyve-1 (green) and R-Spo3 (red) with nuclear staining with DAPI (blue) was performed. CD45^−^Lyve-1^−^ cells were stained as controls. Scale bar, 10 μm. (**i**) The concentration of R-Spo3 in the cell lysates from 3 × 10^5^ sorted VECs, and LECs was measured using ELISA. Data from one of three independent experiments with similar results are shown as the means ± SE (n = 4/group). *P < 0.05, **P < 0.01.

**Figure 3:**
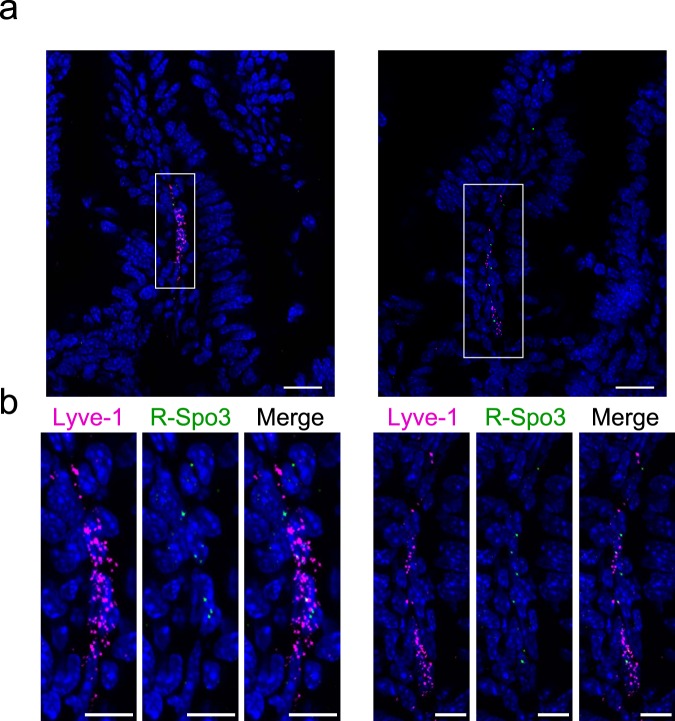
R-Spo3 transcription is detected in LECs by FISH. FISH was performed on the intestinal sections using fluorochrome-labeled probes against *Lyve-1* (magenta) and *R-Spo3* (green) mRNA. (**a**) Representative images from two different parts of the small intestine are shown. (**b**) Images of the Lyve-1 and R-Spo3 signals in the white rectangles on the top panels were magnified and presented as separated or merged images bellow the original images.

**Figure 4:**
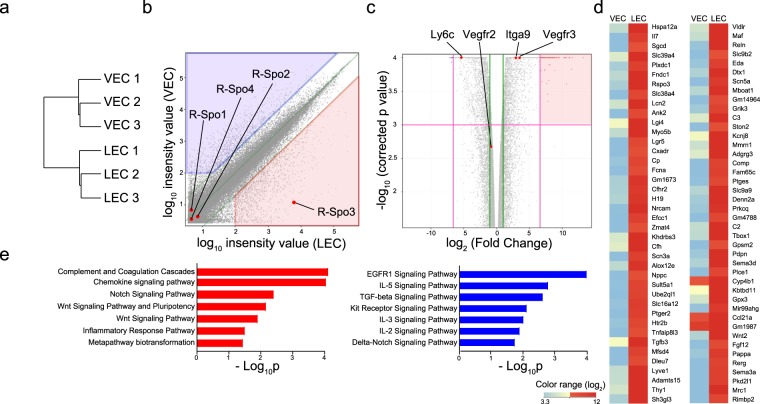
Transcriptome analysis of intestinal LECs. The transcriptome of VECs and LECs sorted from the small intestines was analyzed (n = 3/group). (**a**) Hierarchical clustering using the Euclidean distance of each samples. (**b**) Scatter plot shows the expression levels of each entity in LECs and VECs. The list of 1226 entities that were up-regulated (fold change >5 and expression level in LECs >100; red shaded) and 975 entities that were down-regulated (fold change >5 and expression level in VECs >100; blue shaded) in LECs is shown in Supplementary Table 2. (**c**) Volcano plots with relative differences in expression levels between VECs and LECs and the p value of each entity. Green lines indicate fold change <0.5 or >2, and magenta lines indicate fold change <0.01 or >100 and p < 0.001. (**d**) A heatmap shows the expression levels of highly LEC-specific genes (fold change >100 and p < 0.001; red shaded in Fig. 3c). (**e**) Biological pathways from WikiPathways were assigned to each entity selected in (**b**). The bar charts display the negative log of the enrichment p values for each pathway enriched in LECs (red) or VECs (blue).

To explore the physiological producers of R-Spo3 in the small intestine, we measured expression levels of *R-Spos* in three cell types in the small intestine: intestinal epithelial cells (IECs), CD45^+^ hematopoietic cells, and CD45^−^ non-IEC non-hematopoietic cells. We found that *R-Spo1-3*, but not *R-Spo4*, were detected in the CD45^−^ non-IEC non-hematopoietic cell fraction, with *R-Spo3* expressed at the highest level (Fig. 2a). As previously reported, the CD45^−^ non-IEC non-hematopoietic cell fraction in the intestine includes CD90^+^CD31^−^ mesenchymal cells, such as muscular cells and fibroblasts, CD90^−^CD31^+^ blood vascular endothelial cells (VECs), CD90^+^CD31^+^ lymphatic endothelial cells (LECs), and CD90^−^CD31^−^ other cells (Fig. 2b)^11,12^. Flow cytometric analysis demonstrated that CD45^−^CD90^+^CD31^+^ cells expressed the endothelial marker Tie-2 and lymphatic makers, such as Lyve-1 and Podoplanin, confirming that these cells are LECs (Fig. 2c). In contrast, VECs lack lymphatic markers, but are positive for Gr-1 (Ly-6C/Ly-6G) and Tie-2^13^. We found that most of the CD45^−^Lyve-1^+^ LECs expressed both CD31 and CD90, confirming that the CD45^−^CD31^+^CD90^+^ population is the representative population of intestinal LECs (Fig. 2d). To evaluate the expression of *R-Spos* in LECs, quantitative PCR was performed using RNA from four sorted populations based on CD31 and CD90 expression, as shown in Supplementary Figure 2. We found that the expression level of *R-Spo3* was highest in CD45^−^CD90^+^CD31^+^ LECs, while there was minimal expression of R-Spo1, 2, and 4 in each cell population (Fig. 2e, and data not shown). To study the location of CD45^−^CD90^+^CD31^+^ LECs in the intestine, cells from the lamina propria and serosal layer were separately analyzed using a flow cytometry. We found that the majority of these LECs resided in the lamina propria of the small intestine, while the serosal layer was devoid of LECs (Fig. 2f,g). To confirm R-Spo3 protein production in LECs, LECs were sorted and spun onto glass slides. Immunofluorescent staining demonstrated that R-Spo3 was positive in the nucleus and cytoplasm in LECs with moderate enrichment in the nucleus, but not detected in control CD45^−^Lyve-1^−^ cells (Fig. 2h). Furthermore, ELISA showed that the lysates from sorted LECs contained significantly higher R-Spo3 protein levels than those of VECs (Fig. 2i). *In vivo* R-Spo3 expression in LECs was evaluated by fluorescent *in situ* hybridization (FISH) using fluorochrome-conjugated probes against Lyve1 and R-Spo3 transcripts on the intestinal sections. We confirmed that most of the Rspo3 signals were detected in Lyve-1-expressing LECs in the lamina propria of the small intestine (Fig. 3). Altogether, we concluded that LECs in the lamina propria of the small intestine are the major producers of R-Spo3 in adult mice. To further characterize LECs in the intestine, transcriptome analyses of VECs and LECs were performed using microarrays. Hierarchical clustering using the Euclidean distance of each sample demonstrated that VECs and LECs had distinctive transcriptional profiles (Fig. 4a). In total, 1226 entities up-regulated more than 5-fold in LECs and 975 entities down-regulated more than 5-fold in LECs were selected and are listed in Supplementary Table 2 (Fig. 4b). As expected, highly LEC-specific genes that were defined as fold change >100 and p value <0.001 included well-recognized LEC markers, such as *Pdpn*, *Lyve1*, *and Reln* (Fig. 4c,d). In addition, this category also included growth factors such as *R-spo3*, *Wnt2*, and *Fgf12*, and, interestingly, an R-Spo receptor, *Lgr5*, suggesting that R-Spo3 may have biological effects on LECs by functioning in an autocrine fashion. Furthermore, *Vegfr3* and *Itga9* were specifically expressed in LECs, *Ly6c* was specifically expressed in VECs, and *Vegfr2* was expressed in both populations, further confirming the identity of VECs and LECs (Fig. 4c). When biological pathways were assigned to 1226 entities up-regulated in LECs, WNT signaling pathways were significantly enriched in LECs, suggesting that R-Spo3 may have biological effects on LECs by functioning in an autocrine fashion (Fig. 4e). Although multiple pathways other than WNT signaling pathways were also enriched in LECs and VECs, biological roles of these pathways remain to be clarified.

### R-Spo3 producing LECs are reduced in acute GVHD

**Figure 5:**
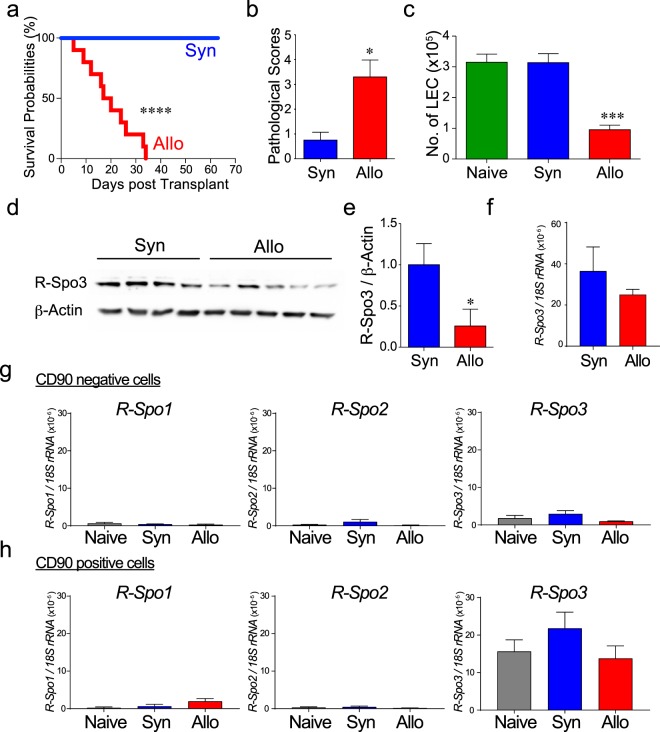
R-Spo3-producing LECs are reduced in acute GVHD. B6D2F1 mice conditioned with busulfan and cyclophosphamide were transplanted with bone marrow cells and splenocytes from allogeneic C57BL/6 (Allo) or syngeneic B6D2F1 (Syn) donors. (**a**) The survival probabilities of the recipients (n = 10/group). (**b**–**f**) The small intestines were harvested on day +14 after transplantation. (**b**) Pathological scores of the small intestines from Syn (n = 4) and Allo mice (n = 6) are shown. (**c**) The absolute numbers of LECs in the small intestine were evaluated by flow cytometric analysis (Naïve; n = 10, Syn; n = 12, Allo; n = 13). (**d**,**e**) R-Spo3 protein in small intestinal lysates was detected by western blotting (**d**), and relative concentrations of R-Spo3 to those of β-actin in the small intestines are shown (**e**). (**f**) Expression levels of *R-Spo3* in LECs from Syn (n = 4) or Allo (n = 5) recipients were measured by quantitative PCR. (**g**,**h**) Expression levels of *R-Spos* in CD45^−^CD90^−^ cells (**g**) and CD45^−^CD90^+^ cells (**h**) were measured by quantitative PCR (n = 4/group). (**a**,**c**,**e**) Data from two similar experiments were combined. (**b**,**f**–**h**) Representative results from one of two independent experiments with similar results are shown. Data are shown as the means ± SE.

Next, we explored the fate of LECs after experimental SCT. B6D2F1 (H-2^b/d^) mice conditioned with a combination of busulfan and cyclophosphamide were transplanted with bone marrow cells and splenocytes from allogeneic C57BL/6 (B6: H-2^b^) or syngeneic B6D2F1 donors. GVHD was severe and all allogeneic recipients died by day +35 after SCT (Fig. 5a). Significant pathological changes caused by acute GVHD were confirmed in the intestine on day +14 after SCT, including surface erosion, ulceration, epithelial apoptotic bodies, lymphocytic infiltration, neutrophilic infiltration, and edema (Fig. 5b). Immunofluorescent staining demonstrated that Lyve-1^+^ lymphatic vessels were reduced in the small intestine from allogeneic mice compared to those from naïve and syngeneic controls (Supplementary Figure 3a). Flow cytometric analysis showed that LECs were persistently reduced during the first 4 weeks after allogeneic SCT compared to syngeneic and naïve mice (Fig. 5c and Supplementary Figure 3b). In association with the reduction of LECs in GVHD, protein levels of R-Spo3 in the small intestine were significantly decreased in allogeneic mice compared to those in syngeneic recipients (Fig. 5d,e). The expression levels of R-Spo3 in LECs on a per cell basis were comparable in syngeneic and allogeneic recipients, indicating that reduced expression of R-Spo3 was merely a reflection of reduced numbers of LECs (Fig. 5f). Finally, we confirmed that there was no aberrant expression of R-Spos in the intestines after syngeneic or allogeneic SCT, indicating that R-Spo3 is the major R-Spo protein produced in the intestine both at steady state and inflammatory conditions (Fig. 5g,h).

## Discussion

R-Spos engage LGR4, LGR5, LGR6, RNF43, and ZNRF3 receptors and potentiate canonical Wnt/β-catenin signaling by inhibiting ubiquitination of WNT receptors^14^. In the intestine, R-Spos in collaboration with WNT ligands play a critical role in the maintenance of tissue homeostasis. However, only R-Spos stimulate ISCs to self-renew, while WNT ligands alone fail to induce self-renewal of ISCs or promote tissue repair after gut injury^14^. R-Spos are indispensable for growth of gastrointestinal organoids *in vitro*, further emphasizing the critical role of intrinsic R-Spos in the maintenance of gut mucosal homeostasis^1,15,16^.

Several previous studies have shown that stromal cells in the intestines produced R-Spo2 and R-Spo3 and support intestinal organoid culture^16–20^. Myofibroblasts isolated from neonatal mice produced R-Spo2 and supported gut organoid generation^18,19^. However, the origin and precise profiles of *in vivo* producers of R-Spos in the adult intestines remained to be investigated. In the current study, we dissected intestinal stromal cells into four populations, namely, CD90^+^CD31^−^ mesenchymal cells, CD90^+^CD31^+^ LECs, CD90^−^CD31^+^ VECs, and CD90^−^CD31^−^ other cells, and found that LECs are the major producer of R-Spo3 in the intestine. In contrast to previous studies^18,19^, the CD90^+^CD31^−^ mesenchymal cell fraction including myofibroblasts did not produce R-Spos in our study. Myofibroblasts may lose the ability to produce R-Spo2 in adulthood. It has also been shown that CD45^−^MYH11^+^α-SMA^+^CD31^−^ mesenchymal cells in the stomach, heart, and liver endothelial cells, possibly including LECs produced R-Spo3^21–23^. Cell types that produce R-Spos may be age and tissue-dependent. Interestingly, Lgr5 is highly expressed in LECs, suggesting that R-Spo3 may have autocrine stimulatory activity on LECs themselves. Our immunofluorescent images showed that R-Spo3 was localized both in the nucleus and cytoplasm in LECs, with moderate enrichment in the nucleus. It has been shown that R-Spo1 mainly localizes to the cytoplasm of quiescent muscular stem cells, while nuclear expression of R-Spo1 is dramatically upregulated after activation of these cells, suggesting that localization of the R-Spos may be context-dependent and that R-Spos translocate between the nucleus and cytoplasm^24^.

Due to the marked hydrophobicity of WNT ligands, they need to exist in close proximity to the responder cells. In the stomach, R-Spo3 preferentially promotes growth of the gastric mucosal epithelial cells residing near the R-Spo3-producing mesenchymal cells, suggesting that mesenchymal cells surrounding the gastric crypt form a niche for gastric stem cells and that R-Spo3 serves as a local proliferative factor of epithelial cells^21^. Recently, Lgr5-positive stromal cells in the lung were shown to form the differentiation niche of mucosal progenitor cells of the alveolar epithelium^25^. The role of lymphatic endothelial cells as a niche for ISCs and the effects of Lgr5 stimulation on LECs need to be elucidated in future studies.

LECs play a critical role in maintaining gut homeostasis. Conditional depletion of LECs in LEC-specific diphtheria toxin receptor-expressing mice causes lethal sepsis, suggesting the critical role of LECs in mucosal barrier function^26^. Stimulation of lymphatic function by vascular endothelial growth factor-C ameliorated inflammatory bowel disease in mice^27^. We found that the numbers of LECs and production of R-Spo3 were significantly reduced in GVHD. Since R-Spos, but not WNT ligands promote tissue regeneration after injury in the intestine and increase Paneth cells^4,9,14^, loss of LECs and R-Spo3 in the intestine could be causally related to GVHD exacerbation. Indeed, R-Spo3 neutralization and to a much lesser extent R-Spo2 neutralization after intestinal injury impaired tissue regeneration, indicating the critical role of intrinsic R-Spo3 production in the homeostasis of gut tissues^10^. However, our results are in sharp contrast to a recent report showing that lymphatic vessels were increased in the intestine in GVHD and that inhibition of lymphangiogenesis ameliorated murine GVHD^28^. We enumerated LECs by flow cytometry, while the previous study used histological analysis in combination with immunohistochemistry of Lyve-1. We also performed immunofluorescent studies of Lyve-1 and found that lymphatic vessels seemed to decrease after allogeneic SCT. However, technical difficulty to make appropriate longitudinal sections of lymphatic vessels hampers us to accurately quantify LECs on the sections, suggesting that flow cytometric analysis is more reliable for quantification of LECs. The dynamics of LECs after SCT could be dependent on severity of GVHD, type of conditioning, HLA disparity, and time course from SCT, requiring further studies.

We previously showed that GVHD targets ISCs and Paneth cells, leading to an impaired intestinal homeostasis and microbial ecosystem, which further exacerbates GVHD^4,29^. Administration of recombinant human R-Spo1 protected Lgr5^+^ ISCs and Paneth cells against GVHD, resulting in maintenance of the integrity of the gut mucosa and intestinal commensals after murine allogeneic SCT^4,9^. Administration of R-Spo1 also ameliorated radiation colitis and experimental inflammatory bowel diseases^30,31^. In this study, we demonstrate a novel role of LECs as the major producer of R-Spo3 in the intestine. A reduction of LECs in GVHD may be associated with delayed tissue repair and defective mucosal defense in intestinal GVHD. The protection of R-Spo-producing LECs or stimulation of LECs to produce R-Spo3 could be potential therapeutic modalities of GVHD.

## Materials and Methods

### Mice

C57BL/6 (B6: H-2^b^) and B6D2F1 (H-2^b/d^) mice were purchased from Japan CLEA (Tokyo, Japan). Animals were allocated randomly for each experimental group, ensuring that the mean body weight in each group was similar. Experiments in the current manuscript were performed in a non-blinded fashion. The Animal Care and Research Advisory Committee of Hokkaido University approved all animal procedures used in this study. The protocol for these experiments was reviewed and approved by the Institutional Animal Care and Research Advisory Committee of Hokkaido University (no. 17–0026). The experimental methods were performed according to the regulations and guidelines established by the Animal Care and Research Advisory Committee of Hokkaido University.

### SCT

Female B6D2F1 mice (8–12 weeks old) were intraperitoneally injected with 25 mg/kg of busulfan (Otsuka, Tokyo, Japan) from day −7 to day −4 and 100 mg/kg of cyclophosphamide (Shionogi, Osaka, Japan) on days −3 and −2 followed by intravenous injection with 1 × 10^7^ splenocytes and 5 × 10^6^ bone marrow cells from allogeneic B6 or syngeneic B6D2F1 donors on day 0, as previously described^32^. Mice were maintained in specific-pathogen-free conditions and received normal chow and autoclaved hyperchlorinated water for the first 3 weeks after SCT and filtered water thereafter. Survival after SCT was monitored daily.

### Flow cytometry and cell sorting

Cell suspensions of the small intestine were prepared as previously described^33^. After removal of Peyer’s patches, small intestines were incubated on a shaker in PBS containing 2% FBS (complete medium; CM) and 1 mM DTT (Sigma-Aldrich, St. Louis, MO) at 37 °C for 20 minutes and subsequently incubated with 1.3 mM EDTA (Nippon Gene, Tokyo, Japan) in CM at 37 °C for 1 hour. After harvesting the supernatants containing IECs, the rest of the intestinal specimens were digested with 0.3 mg/ml type IV collagenase (Sigma-Aldrich) at 37 °C for 1 hour, homogenized, and filtered. To separate serosal cells and lamina propria cells, the intestinal muscularis covered by the serosa was peeled off before digestion using fine surgical forceps and a dissection microscope^34^. The cell suspension was stained with antibodies listed in Supplementary Table 1. Dead cells were removed from the analyses as DAPI (Dojindo Laboratories, Kumamoto, Japan)-positive cells. Cells were analyzed with a FACS CantoII (BD Biosciences, San Jose, CA). Fluorescence-activated cell sorting was performed using a FACS Aria II (BD Biosciences).

### Cytospin

Cell suspensions from murine small intestines were stained with anti-Lyve-1 and anti-CD45 monoclonal antibodies. CD45^−^Lyve-1^+^ LECs and CD45^−^Lyve-1^−^ cells were spun onto glass slides, and dried overnight. The slides were steeped in methanol for 10 minutes at −20 °C and acetone for 1 minute. The slides were washed with PBS and blocked in 0.1% BSA for 30 min. The cells were stained with anti-R-Spo3 rat monoclonal antibodies (R&D Systems, Minneapolis, MN) and anti-Lyve-1 polyclonal rabbit antibodies (AngioBio, San Diego, CA), and then incubated for 1 hour at room temperature with the Alexa Fluor 488-conjugated goat anti-rabbit IgG antibodies and Alexa Flour 555-conjugated goat anti-rat IgG antibodies. Nuclear staining was performed with 1 μg/mL of DAPI. Images were acquired at room temperature using a Fluoview FV1000 confocal microscope (Olympus, Tokyo, Japan) with 60×/1.35 numerical aperture (NA).

### Immunofluorescence

Tissue samples were fixed with 4% paraformaldehyde overnight and embedded in paraffin. After antigens retrieval was performed Target Retrieval Solution pH 6 (Dako Japan, Kyoto, Japan) at 105 °C for 20 min, the sections were treated with blocking reagents containing 0.1% bovine serum albumin for 30 min, followed by incubation with rabbit anti-mouse Lyve-1 antibody at 4 °C overnight. The primary Abs were visualized by incubating with Alexa Fluor 488-conjugated goat anti-rabbit antibody for 1 h, and nuclei were stained with DAPI.

### FISH

FISH was performed using the Quantigene View RNA ISH Cell Assay (Affymetrix, Santa Clara, CA, USA) and following the manufacturer’s instructions with slight modifications. Briefly, the fresh frozen sections of the small intestine were fixed in 4% paraformaldehyde in PBS for 30 min and then washed three times in 50 mM glycine in PBS. The sections were pretreated with a detergent solution for 10 min and then 0.1 mg/ml proteinase K in PBS (Kanto Chemical co, Tokyo, Japan) for 10 min at room temperature. Following processes were performed in accordance with the manufacturer’s instruction. Specific oligonucleotide probe sets against *Rspo3* (Assay ID: VB1-11826) and *Lyve1* (Assay ID: VB6-18604) were purchased from Affymetrix, Inc.

### Quantitative PCR

Total RNA extracted from the small intestine was subjected to conventional reverse transcription by using ISOGEN II (Nippon Gene). cDNA was synthesized using ReverTra Ace qPCR RT Master Mix with gDNA Remover (FSQ-301; Toyobo, Osaka, Japan). Quantitative PCR was performed on the ABI StepOnePlus System (Applied Biosystems, Foster City, CA) using TaqMan Fast Advanced Master Mix (Applied Biosystems) and primer-probe sets (Sigma Aldrich). The *18S rRNA* primer probe set was 5′-GCTCTTTCTCGATTCCGTGGG-3′ for the forward primer; 5′-ATGCCAGAGTCTCGTTCGTTATC-3′ for the reverse primer, and FAM-CTCCACCAACTAAGAACGGCCATGCACC-TAMRA for the probe. The *R-Spo1* primer-probe set was 5′-CGCAACCCCGACATGAACA-3′ for the forward primer, 5′-GACACTTGGTGCAGAAGTTGTG-3′ for the reverse primer, and FAM-CCTCACAGTGCTCGATCTTGCATT-TAMRA for the probe. The *R-Spo2* primer-probe set was 5′-GCCGCTGCTTTGATGAATGTCC-3′ for the forward primer, 5′-CTCCAATGACCAACTTCACAACCTT-3′ for the reverse primer, and FAM-ACACATTCCATAGTCTCATCTAACGGTGCA-TAMRA for the probe. The *R-Spo3* primer probe set was 5′-AACGCCTCCCGAGGAAGG-3′ for the forward primer, 5′-ACAGCCCATTGTAATCTGAACACG-3′ for the reverse primer, and FAM-GCCTCCTTGGCAGCCTTGACTGACA-TAMRA for the probe. The *R-Spo4* primer-probe set was 5′-TTGCCGAGTGTTATCTGAGTCC-3′ for the forward primer, 5′-CGCCGATCCTTCCTGTTCTTC-3′ for the reverse primer, and FAM-ACTCTGCCCAGGAGAAAGAAATCCCAGG-TAMRA for the probe. The relative expression of target genes was determined by the comparative *Δ*C(t) method. Expression levels of *18S rRNA* were used as a standard.

### Western blotting

The small intestine was lysed in RIPA buffer (50 mM Tris–HCl (pH 8.0), 150 mM NaCl, 1% NP-40, 0.1% SDS, 0.5% sodium deoxycholate, 5 mM EDTA, 0.1% Triton X-100) supplemented with 50 mM N-ethylmaleimide, 10 μg/ml aprotinin, 50 μg/ml leupeptin, 100 mM NaF and 1 mM Na3VO4. Western blotting was performed as previously described^35^. Proteins were separated by SDS-PAGE and transferred onto nitrocellulose membranes and detected with antibodies shown in Supplementary Table 1. Immunoblot signals were detected using ECL Prime Western Blotting Detection Reagent and the ImageQuant LAS4000 mini system (GE Healthcare, Buckinghamshire, UK), and the band intensity was quantified using ImageJ software (http://imagej.nih.gov/ij/). To detect R-Spo1, R-Spo2, and R-Spo3 separately, gels were cut into three parts and each part of the gel was incubated with antibodies to R-Spo1, R-Spo2, or R-Spo3, respectively. All parts of the gel were gathered again after washing and the image of whole gel was taken. Then, the gel was incubated with anti-β-actin antibodies and detected with the shorter exposure time than that for R-Spondins. The molecular weights of R-Spo1, R-Spo2, and R-Spo3 are 29 kDa, 28 kDa, and 31 kDa, respectively. Recombinant mouse R-Spo3 (R&D Systems) was used as a control.

### ELISA

The levels of R-Spo3 in the lysates from 3 × 10^5^ sorted VECs and LECs were measured using a Mouse R-Spondin 3 DuoSet ELISA kit (R&D systems) according to the manufacturer’s instructions with a sensitivity of 9.38 pg/ml.

### Microarray

LECs and VECs were sorted from the small intestine. Total RNA was extracted using Trizol reagent (Thermo Fisher Scientific, Yokohama, Japan) and QIAshredder column (QIAGEN) according to the manufacturer’s instructions. Cyanine-3 (Cy3)-labeled cRNA was prepared from 0.1 μg of total RNA using a Low Input Quick Amp Labeling Kit (Agilent technologies, Foster City, CA) according to the manufacturer’s instructions and hybridized to a SurePrint G3 Mouse GE 8 × 60 K Ver2.0 (Agilent) for 17 hours at 65 °C. After hybridization, slides were scanned immediately after washing on the Agilent SureScan Microarray Scanner (G2600D) using one color scan setting for 8 × 60k array slides. Data were preprocessed, normalized, and further analyzed using Gene Spring GX software (Agilent technologies). ANOVA was used for the selection of differentially expressed genes among target populations. Pathway analysis was performed using WikiPathways (https://www.wikipathways.org/index.php/WikiPathways). Microarray data were deposited in the Gene Expression Omnibus database (GEO, https://www.ncbi.nlm.nih.gov/geo/, accession number: GE104979).

### Statistical analysis

Mann-Whitney U tests were used to compare data, the Kaplan-Meier product limit method was used to obtain survival probability, and the log-rank test was applied to compare survival curves. All tests were performed with the statistical program Prism (GraphPad, San Diego, CA). *P* < 0.05 was considered statistically significant.

### Data availability

The datasets analyzed during the current study are available from the corresponding author upon reasonable request.

## Electronic supplementary material

dataset1

dataset2
