# Supplementary material for: Intestinal Lymphatic Endothelial Cells Produce R-Spondin3
Source: Sci Rep. 2018 Jul 16;8:10719. doi: 10.1038/s41598-018-29100-7 (PMC6048029; doi:10.1038/s41598-018-29100-7)
Supplement: Supplementary file 1 — dataset1 [file 41598_2018_29100_MOESM1_ESM.pdf]

Supplemental Information

Intestinal Lymphatic Endothelial Cells Produce R-Spondin3

Reiki Ogasawara, Daigo Hashimoto, Shunsuke Kimura, Eiko Hayase, Takahide Ara, Shuichiro Takahashi, Hiroyuki Ohigashi, Kosuke Yoshioka, Takahiro Tateno, Emi Yokoyama, Ko Ebata, Takeshi Kondo, Junichi Sugita, Masahiro Onozawa, Toshihiko Iwanaga, Takanori Teshima

Supplementary Legends

Supplementary Figure 1. R-Spo3 is the major R-Spo molecule produced in the intestine.

Western blotting analyses to detect R-Spo1, R-Spo2, and R-Spo3 in the lysate of small intestine were performed as Fig. 1b. Images of full-length gel were taken with 1, 2, 3, or 5 minutes exposure time. rmR-Spo3 was used as a size control.

Supplementary Figure 2. Sorting strategies for LEC and VEC.

Cells harvested from small intestine were labeled with fluorochrome-conjugated mAbs to CD45, CD90, and CD31. Doublets and dead cells were excluded as FSC-W^{high} and $DAPI^{+}$ cells, respectively. Cells within the red gates were shown on the right of the original dot plots.

Supplementary Figure 3. Immunofluorescent staining of lymphatic vessels in the small intestine after SCT.

Mice were transplanted as in Fig. 5, and the small intestines were harvested on day +14 after transplantation. (**a**) Immunofluorescent images of Lyve-1 (green) with DAPI nuclear staining (blue) were shown. Bar, 100 μm. (**b**) LECs were enumerated using flow cytometry on days +14 and +28 after syngeneic SCT and on days +7, +14, and +28 after allogeneic SCT (Allo). Data from two independent experiments were combined and shown as means ± SE (n = 5-8 / group). $^{*}P<0.05$, $^{**}P<0.01$.

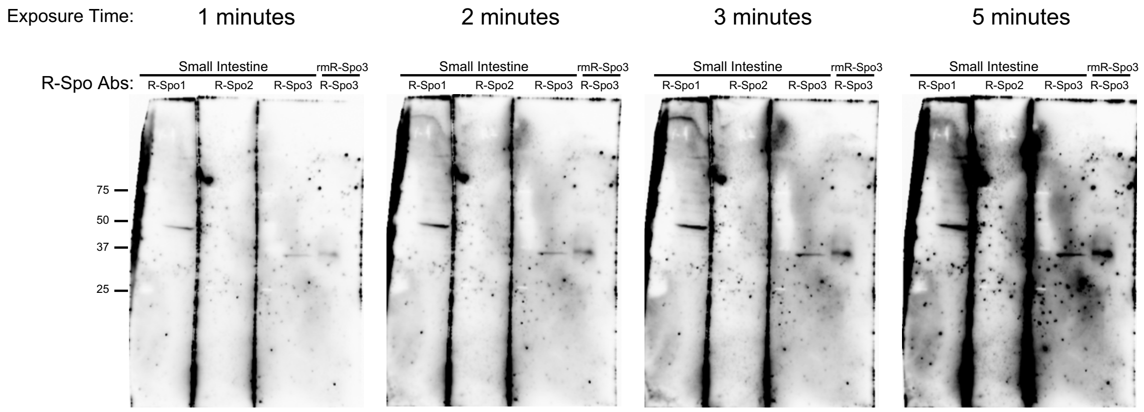

Supplementary Figure 1.

Ogasawara, et al.

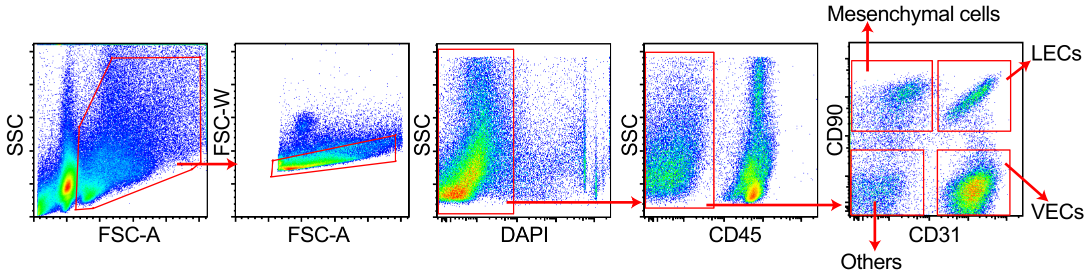

Supplementary Figure 2.

Ogasawara, et al.

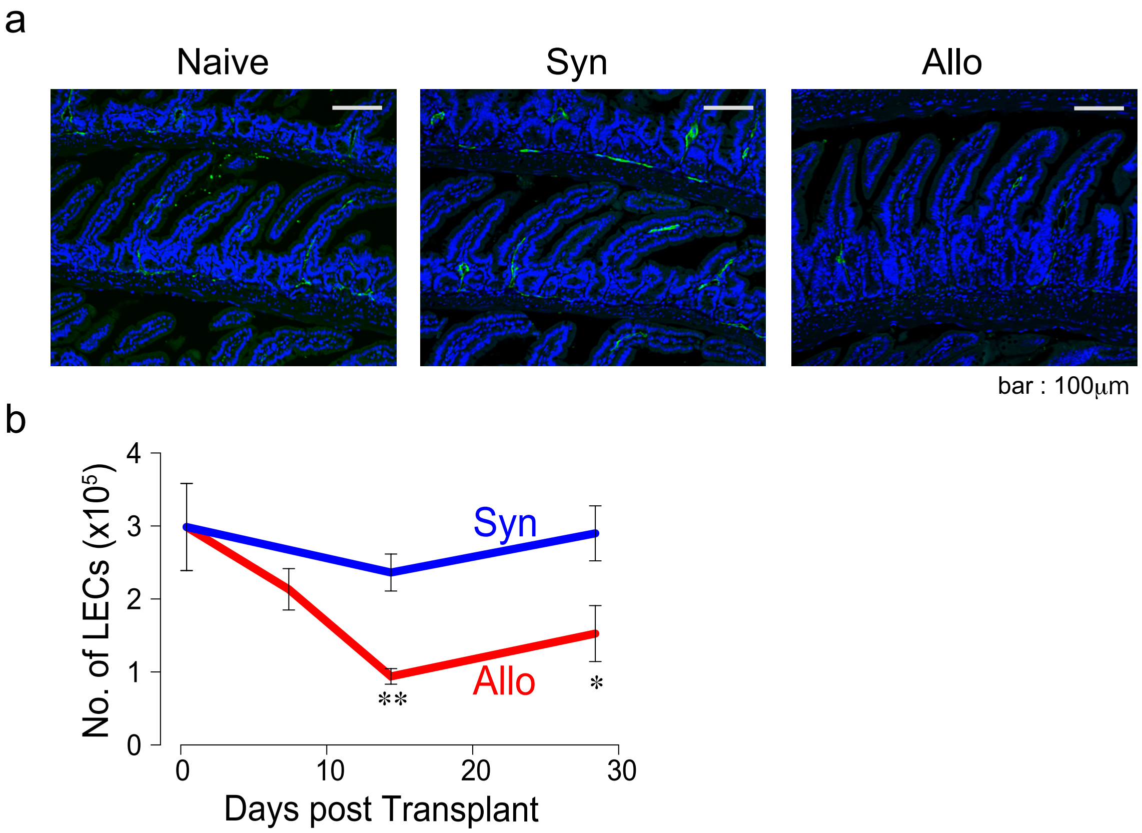

Supplementary Figure 3.

Ogasawara, et al.

Supplementary Table 1. List of primary antibodies used in immunofluorescent studies, flow cytometry, and Western blotting

Target Ag	Clone	Conjugate	Supplier	Catalog#
Flow cytometry				
CD45	30-F11	APC-Cy7	BD Bioscience	557659
CD90.2	53-2.1	PerCP/Cy5.5	Biolegend	140322
CD31	390	PE-Cy7	eBioscience	25-0311-82
I-A/I-E	M5/114.15.2	FITC	Biolegend	107606
CD4	GK1.5	PE	BD Pharmingen	553730
CD8	53-6.7	PE	BD Bioscience	553033
F4/80	BM8	FITC	Biolegend	123108
CD11b	M1/70	FITC	BD Pharmingen	01714D
CD11c	HL3	FITC	BD Pharmingen	553801
CD103	2E7	PE	BioLegend	121406
Ly6C/Ly6G	RB6-8C5	FITC	BD Bioscience	553127
E-Cadherin	DECMA-1	PE	BioLegend	147304
Tie2	TEK4	Biotin	BioLegend	124006
Lyve-1	polyclonal	purified	AngioBio	11-034
Podoplanin	8.1.1	PE	BioLegend	127408
Immunofluorescence				
Lyve-1	polyclonal	purified	AngioBio	11-034
R-Spo3	# 400403	purified	R&D systems	MAB41201
Western blotting				
R-Spo1	polyclonal	purified	abcam	ab106556
R-Spo2	polyclonal	purified	abcam	ab73761
R-Spo3	polyclonal	purified	abcam	ab171010
R-Spo3	polyclonal	purified	abcam	ab109808
β-actin	AC1-20.4.2	purified	Sigma-Aldrich	A9357
